# Kaposi’s sarcoma-associated herpesvirus protein ORF75 among HIV-1 patients in Kenya

**DOI:** 10.4102/ajlm.v9i1.939

**Published:** 2020-08-25

**Authors:** Rodgers N. Demba, Sylviah M. Aradi, Matilu Mwau, Walter O. Mwanda

**Affiliations:** 1 School of Health Sciences, Kisii University, Kisii, Kenya; 2 Institute of Tropical and Infectious Diseases, University of Nairobi, Nairobi, Kenya; 3 Department of Internal Medicine, University of Nairobi, Nairobi, Kenya; 4 Center for Infectious and Parasitic Diseases Control Research, Kenya Medical Research Institute, Busia, Kenya

**Keywords:** Human herpes virus 8, Kaposi’s sarcoma, histology, nested PCR, ORF75 gene

## Abstract

**Background:**

Histology is used to identify Kaposi’s sarcoma (KS) in countries with low resources to fund healthcare costs. Approximately 95% of KS cases can be detected using a polymerase chain reaction.

**Objective:**

To determine the presence of the open reading frame 75 (ORF75) gene associated with Kaposi’s sarcoma herpes virus among HIV-1/AIDS patients and to describe morphological presentations of KS.

**Methods:**

This was a retrospective, descriptive study of archived tissue blocks collected from 2013 to 2016. Haematoxylin and eosin staining was used to identify KS. Deoxyribonucleic acid from archived tissue blocks was extracted and a nested polymerase chain reaction was used to detect the ORF75 gene.

**Results:**

All 81 cases in this study had been diagnosed as HIV-1 positive, of which 68 had hallmark features of KS in the histology report and 13 had features suggestive of KS (‘KS-like’). Microscopic identification of KS by haematoxylin and eosin staining was considered a significant indicator of KS herpes virus ORF75 gene positivity (*p* = 0.002). The ORF75 gene was detected in 60.5% (49/81) of tissue blocks; 27.2% were men (22/81) and 33.3% were women (27/81). The ORF75 gene was observed to be present in up to 15.4% (2/13) of the cases reported to have KS-like features.

**Conclusion:**

Following the initial diagnosis of KS by histology, the ORF75 gene was fur-ther detected from both cases that had hallmark features of KS as well as among cases with KS-like fea-tures.

## Introduction

Kaposi sarcoma (KS) is a tumor formed from blood vessels; it later shows lesions on the skin or organs of HIV-positive people.^1^ All forms of KS are caused by Kaposi’s sarcoma herpesvirus (KSHV), also known as human herpesvirus 8 (HHV-8).^2,3^ The genome of HHV-8 contains a minimum of 100 open reading frames (ORF), of which 4 to 75 are known to be unique to herpesvirus.^4^ The KSHV genome encodes more than 84 proteins that play a role in viral replication and host-virus interaction.^5^ The replication cycle of KSHV entails latent and lytic phases. During the lytic cycle, the ORF75 genes are expressed resulting in the manifestation of KS.^6,7^ The ORF75 gene product has been proven to aid in lytic replication and enhancement of virus pathogenesis in host cells.^8^

Kaposi’s sarcoma is listed among the defining malignancies of HIV/AIDS.^9,10,11^ The dis-tinct feature of HIV-associated KS is that it might affect the lymph nodes, gastrointestinal tract, lungs or liv-er.^12,13^ Despite the fact that saliva is the main route by which KSHV is transmitted,^14^ HHV-8 has been isolated from other body fluids.^14,15,16^ The main route of HHV-8 transmission to the opposite sex is through sexual relations.^17^ The pathogenesis of KS presents as an abnormal neoangiogenesis, proliferation of cancer cells and inflammation of endothelial cells.^18^ A classic KS lesion manifests various features ranging from maculopapular to nodular or plaque-like and, in most cases, is pain-less.^12,19,20,21^

In sub-Saharan Africa, KSHV is endemic and approximately 84% of worldwide cases of KS occur in this region.^22^ Since KS is common among HIV/AIDS pa-tients,^13^ early detection of KSHV is essential in disease monitoring.^22^ The sensitivity of diagnostic tests for detection of KSHV depends on the sample selected for analysis.^23^ For example, biopsies obtained from patients with HIV/AIDS-KS were found to yield better results compared to using peripheral blood mononuclear cells from the same patient.^23^ Identification of KS in tissue biopsies by use of histological staining techniques should not be underestimated.^24^ In tissue biopsies, microscopic examination involves identification of proliferated spindle cells and oedema.^25^ Clinical diagnoses of KS have been shown to have limited predictive value.^26^ The use of molecular techniques such as polymerase chain reaction (PCR) permits the detection of the HHV-8 gene even for patients who present with early vascular lesions that histological techniques might miss.^27^ The use of PCR in the diagnosis of KS can detect approximately 95% of cases.^28^ The HHV-8 DNA has been successfully amplified using nested PCR previously.^29,30^ This study was aimed at determining the presence of the ORF75 gene linked to KSHV among HIV-1/AIDS patients. In addition, the objective of this current study was to describe the morphological presentations of KS among the studied cases.

## Methods

The present study only included patients aged 18 years and older. Data on clinical information that was useful for this study were extracted from the registry records with the help of the data clerk. The following data were obtained from the registry records: sex, age, HIV-1 status, if patient was on antiretroviral or Highly Active Antiretroviral Therapy treatment, anatomic location of KS lesions, number of KS lesions, distribution of KS lesions, cluster of differentiation 4 cell count and histology diagnosis.

### Ethical considerations

Study approval number P682/11/2014 was assigned by Kenyatta National Hospital/University of Nairobi Ethics and Research Committee.

### Study design

A cross-sectional, descriptive, hospital-based study was used. Formalin-fixed, paraffin-embedded tissue blocks were retrieved from archives following histological reports of the patients who were diagnosed with KS or KS-like disease between 2013 and 2016. A consecutive sampling technique was used to select the archived tissue blocks from Thematic Unit of Anatomic Pathology, Department of Human Pathology, College of Health Sciences, University of Nairobi, and Department of Laboratory Medicine, Cytology Section, Kenyatta National Hospital.

For this study, a total of 81 tissue blocks were selected and analysed. A rotary microtome was used to section the formalin-fixed, paraffin-embedded blocks. A different blade was used for every formalin-fixed, paraffin-embedded block so as to avoid carry-over of genetic material. Once a block was cut, the microtomes surface was decontaminated using DNAZap^TM^ PCR DNA degradation solution (catalog number: AM9890; Thermo Fisher Scientific, Waltham, Massachusetts, United States). Each tissue section was cut to 10 µm thick. The tissue sections were processed for haematoxylin and eosin staining and a qualified pathologist reported on the results.

### Deoxyribonucleic acid extraction and polymerase chain reaction

Isolation of DNA from tissue sections was done using a GeneRead DNA FFPE kit (Qiagen, Hilden, Germany). The extraction kit removes paraffin and reverses formalin cross-links from tissue before DNA is bound to the QIAampMinElute column (Qiagen, Hilden, Germany). The eluted DNA is then ready to be used for nested PCR to detect the ORF75 gene in HHV-8. A Taq PCR Core Kit (catalog number: 201223; Qiagen, Hilden, Germany) was used to detect the ORF75 gene. The set of primers used were; ORF75 product size 895 bp Forward KS 1000 5′CGGTTCGGTGGCATACAGGC3′; Reverse KS 1034 5′CTGACTACAGAGGGTGTCCCCG3′.^31^ ORF75 product size 804 bp Forward KS 2000 5′GGAAACAGGGTGCTGTG3′; Reverse KS 2034 5′CATGGCCTACGACGTCAC3′.^32^ The cycling conditions of the PCR for the targeted KS regions were similar and consisted of 30 cycles of: initial denaturation at 94 °C for 3 minutes, denaturation at 94 °C for 1 min, annealing at 63 °C for 1 min, extension at 72 °C for 1 min and final extension at 72 °C for 10 min. Amplified PCR products were analysed by electrophoresis on a 1% agarose gel containing ethidium bromide (1 *µ*L/mL of agarose solution) and were visualised under ultraviolet light alongside a 1 Kilobase (Kb) deoxyribonucleic acid (DNA) ladder. For a positive control, a known case of KS was used. The ribonuclease-free water was used as a negative control.

### Statistical analysis

The data were analysed using Statistical Package for Social Sciences version 21 (SPSS Inc Binghamton, New York, United States); the relationship between the ORF75 gene and clinical characteristics were tested by using chi-square and *t*-tests. A *p*-value of less than 0.05 was considered to be statistically significant. Odds ratios in a cross-sectional study are known as prevalence odds ratios and were used as a measure of association.^33^

## Results

Of the 81 tissue samples included in the study, 43.2% (35/81) were from women and 56.8% (46/81) were from men (Table 1). All of the 81 cases studied had been diagnosed with HIV-1 implying that they were living with the virus. In addition, it was observed that none of the cases had a cluster of differentiation 4 cell count above 350 cells/mm^3^. Among the 81 cases, the ORF75 gene was detected in 49 cases (60.5%); 27.2% (22/81) were women and 33.3% (27/81) were men. Among cases positive for the ORF75 gene, 4.1% (2/49) were never on any form of antiretroviral therapy and 95.9% (47/49) were on antiretroviral therapy. No statistically significant association was found between the presence of the ORF75 gene and sex, antiretroviral treatment status, number of KS lesions or distribution of the KS lesions (all *p*-values > 0.05).

**TABLE 1:** Crude prevalence odds ratio and 95% confidence intervals for patient characteristics and presence of the KSHV ORF75 gene, Nairobi, Kenya, 2013–2016.

Variables	Characteristics		Positive ORF75 gene		POR	95% CI	P*
	*n*	%	*n*	%			
**Gender**	-	-	-	-	1.2	0.48–2.94	0.70
Male	46	56.8	27	33.3	-	-	-
Female	35	43.2	22	27.2	-	-	-
**Age**	-	-	-	-	1.05	1.00–1.11	0.047*
18–29 years	9	11.1	3	6.1	-	-	-
30–39 years	39	48.1	21	42.9	-	-	-
40–49 years	23	28.4	18	36.7	-	-	-
50–59 years	6	7.4	5	10.2	-	-	-
60 years and above	4	4.9	2	4.1	-	-	-
**On treatment**	-	-	-	-	0.64	0.09–4.78	0.66
On ARVs	77	95.1	47	95.9	-	-	-
HAART naïve	4	4.9	2	4.1	-	-	-
**Number of lesions**	-	-	-	-	0.56	0.23–1.38	0.21
> 10	45	55.6	30	61.2	-	-	-
< 10	36	44.4	19	38.8	-	-	-
**Distribution of lesions**	-	-	-	-	1.23	0.48–3.14	0.66
Generalised	53	65.4	33	67.4	-	-	-
Localised	28	34.6	16	32.6	-	-	-
**Histology diagnosis**	-	-	-	-	12.31	2.51–60.49	0.002*
KS	68	84	47	95.9	-	-	-
KS-like	13	16	2	4.1	-	-	-

ORF, open reading frame; *n*, number; POR, prevalence odds ratio; CI, confidence interval; ARVs, antiretrovirals; HAART, Highly Active Antiretroviral Therapy; KS, Kaposi’s sarcoma; KSHV, Kaposi’s sarcoma herpesvirus.

* , *p*-values of < 0.05 were considered statistically significant.

### Age

The mean age of patients with tissue blocks positive for the ORF75 gene was 41 years (standard deviation = 9.2; maximum age, 66 years; minimum age, 19 years). Detection of the ORF75 gene was most common in the 30–39 years age group (*n* = 21; 42.9%). Age had a statistically significant association with ORF75 gene positivity (prevalence odds ratio: 1.05; 95% confidence interval: 1.00–1.11, *P* = 0.047).

### Kaposi sarcoma morphology and distribution of lesions

**FIGURE 1:**
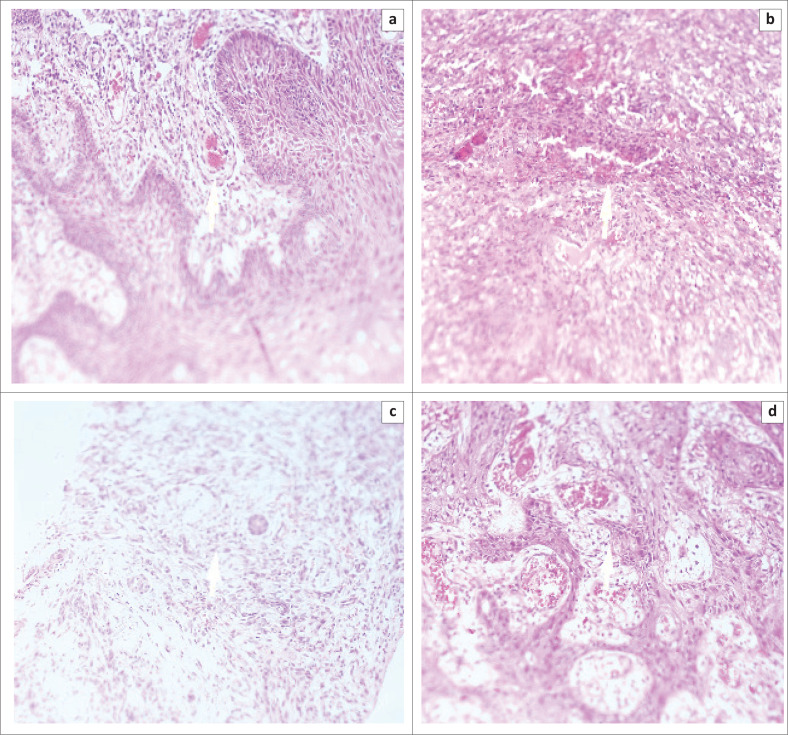
Morphological descriptions of Kaposi’s sarcoma and Ka-posi’s sarcoma-like cases, obtained from University of Nairobi and Kenyatta National Hospital, Nairobi, Kenya, 2013–2016. Microscopic identification by haematoxylin and eosin staining, X20. (a) Patchy, (b) Nodular, (c) Plaque and (d) KS-like.

In the histology report, 68 cases had hallmark features of KS, whereas 13 cases had features suggestive of KS (KS-like). The types of KS morphology identified included patchy, nodular, plaque and KS-like (Figure 1). The morphological distribution of KS was as follows: 61.7% (50/81) was nodular, 16% (13/81) was patchy, and 22% (18/81) were plaques. Among the cases that were positive for the ORF75 gene, 75.51% (37/49) was nodular, 4.08% (2/49) patchy, and 20.41% (10/49) were plaques.

The total number of KS cases diagnosed by histology was 68 (84%) and 13 cases (16%) had KS-like features (Table 1). Among the 49 cases with the ORF75 gene, 47 (95.9%) showed hallmark features of KS and 2 (4.1%) had KS-like features with microscopic examination. There was an association between microscopic identification of KS by histology and the presence of the ORF75 gene (prevalence odds ratio = 12.3; 95% confidence interval = 12.51 – 60.49; *P* = 0.002) (Table 1).

**FIGURE 2:**
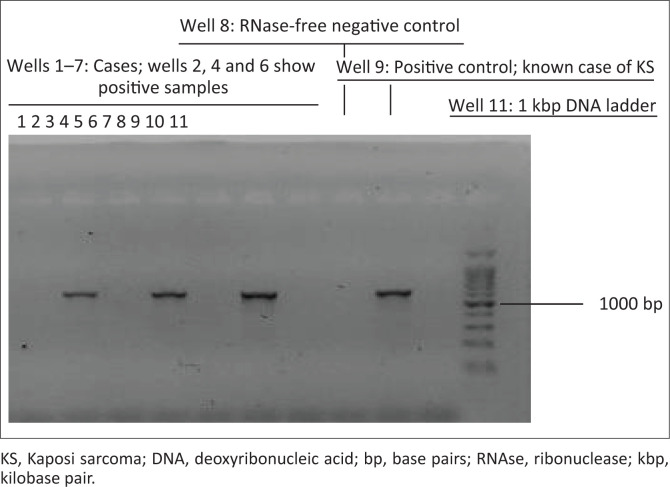
Polymerase chain reaction agarose gel electrophoresis results of Ka-posi’s sarcoma herpes virus ORF75 gene. Cases obtained from University of Nairobi and Kenyatta National Hospital, Nairobi, Kenya, 2013–2016.

The amplified ORF75 genes of HHV-8 were identified by 1% agarose gel electrophoresis (Figure 2).

## Discussion

Retrieved clinical data revealed that all of the tissue blocks retrieved in the present study were collected from patients who had been diagnosed with HIV-1. These patients might have developed KS lesions due to immunosup-pression or because they were immunocomprised due to increased viral load that impaired their immune system. Other studies have also associated KS as an HIV/AIDS-defining illness.^9,13,24,34,35,36^

The findings of this study revealed that men were more prone to development of KS: 56.8% (46/81) compared with women 43.2% (35/81). This observation is concordant with others who also noted more frequent development of KS among men.^37,38,39,40^ There is a lack of consensus as to why all forms of KS are more common among men than women.^41,42^ We hypothesise that gender-related factors such as hormones might influence the development of KS lesions. The results of this study showing KS pre-ponderance among men was consistent with the country’s published data on the distribution of malignancy cases as captured in the National Cancer Control strategy, 2017.^43^

Kaposi’s sarcoma immune reconstitution occurs when a portion of AIDS-KS cases responds to the introduction of combined antiretroviral therapy with disease advancement.^44,45,46^ In this study, KS lesions manifested among patients despite the fact that 77 (95.1%) were on antiretroviral treat-ment. Contrary to other findings that antiretroviral therapy alone can result in the resolution of KS,^47,48^ in our study, being on antiretroviral treatment did not have a statistically significant association with the presence of KS (*P* = 0.66). This finding is in agreement with another study that stated that there has been continued diagnosis of KS in HIV-positive patients, despite the availability of highly active antiretroviral therapy.^49^ Other studies have stated that patients infected with AIDS-associated KS respond to combined antiretroviral therapy by 50% depending on geographical location and severity of the presentation, thereby resulting in immune reconstitution and HIV suppression.^50,51,52^ In the current study, these manifestations of KS could be attributed to the weakening of the immune system by HIV-1.

This study found patchy, plaque and nodular morphological presentations of KS. The morphological appearance of KS shows progression from plaques to nodular form and fungiform.^1^ Kaposi sarcoma lesions are known to progress from asymptomatic to macule, papule, plaque and nodule forms.^53^ The findings of this study revealed that the KS lesions were disseminated in different body regions, including the lower limbs, upper limbs, genitalia, eyelids, palate, oral cavity and trunk (chest and back). In another study, fatality was witnessed in HIV-positive patients who had KS lesions manifested in the gastrointestinal tract, lungs and lymph nodes.^54^

The decision in this study to use tissue biopsy for detection of the ORF75 gene of HHV-8 is in agreement with another study that supported the use of tumor biopsies as suitable for viral DNA identification due to high viral load as opposed to the use of blood.^30^ Further to that, nested PCR has been used successfully to assess the prevalence of HHV-8 among HIV-positive patients in Brazil.^27^ A tissue biopsy excised from a KS lesion has been shown to have high viral load; hence, biopsies are the ideal sample for the detection of KSHV DNA.^55^ The detection of the ORF75 gene implies that this gene was present in 49 (60.5%) of the studied cases.

### Strength and limitations

Our study used the haematoxylin and eosin staining technique and the nested PCR method for detection of the ORF75 genes of the KSHV. However, HHV-8 immunohistochemical biopsy has been demonstrated to be the ‘gold standard’ for KS diagnosis.^56^ Cases in the present study had a dark skin pigmentation. In another study of dark-skinned patients, KS had been confirmed to mimic a number of non-KS-like dermatological conditions.^56^ In another study, it was observed that it is difficult to identify KS in dark-skinned individuals, who presented with violaceous skin lesions.^56^

The use of the PCR technique in the detection of KSHV has been shown to give the utmost specificity compared to the use of tests that determine exposure to infection.^28^ In addition, the PCR technique can detect approximately 95% of all KS cases.^28^ However, the cost associated with the use of PCR is quite high, which would limit the clinical application of HHV-8 DNA detection in resource-limited facili-ties.^28^

### Implications and recommendations

The present study considered microscopic detection of KS by haematoxylin and eosin as a significant indicator of KSHV ORF75 gene positivity. It therefore recommends the use of both clinical diagnosis and routine microscopy in the diagnosis of KS in resource-limited facilities. However, among individuals with dark skin pigmentation, there is the need to employ the use of a robust diagnostic technique to ascertain the true causative agent.

### Conclusion

The presence of the ORF75 gene of KSHV among immunosuppressed patients due to HIV-1 was successfully detected. Following the initial diagnosis of KS by histology, the ORF75 gene was further detected from both cases that had the hallmark features KS and those that had KS-like features. Microscopic detection of KS by haematoxylin and eosin should be considered a significant indicator of KSHV ORF75 gene positivity.
